# Genome Re-Annotation and Transcriptome Analyses of *Sanghuangporus sanghuang*

**DOI:** 10.3390/jof9050505

**Published:** 2023-04-23

**Authors:** Zi-Qi Shen, Ji-Hang Jiang, Chang-Tian Li, Yu Li, Li-Wei Zhou

**Affiliations:** 1 Internationally Cooperative Research Center of China for New Germplasm Breading of Edible Mushroom, Ministry of Science and Technology, Changchun 130118, China; 2 State Key Laboratory of Mycology, Institute of Microbiology, Chinese Academy of Sciences, Beijing 100101, China; 3 University of Chinese Academy of Sciences, Beijing 100049, China

**Keywords:** *Basidiomycota*, *Hymenochaetales*, medicinal functions, secondary metabolites, wood-inhabiting macrofungi

## Abstract

*Sanghuangporus sanghuang*, the generic type of *Sanghuangporus* belonging to *Hymenochaetaceae*, is a precious medicinal wood-inhabiting macrofungus with high commercial potential. To facilitate the medicinal utilization of this fungal resource, transcriptome sequences are newly generated from *S*. *sanghuang* strain MS2. In association with the previously generated genome sequences from the same strain by our lab and all available fungal homologous protein sequences in the UniProtKB/Swiss-Prot Protein Sequence Database, a new methodology was employed for genome assembly and annotation. A total of 13,531 protein-coding genes were identified from the new version of the genome of *S*. *sanghuang* strain MS2 with a complete BUSCOs of 92.8%, which indicates a remarkable improvement in the accuracy and completeness of the genome assembly. In general, more genes involved in medicinal functions were annotated compared with the original version of the genome annotation, and most of these genes were also found in the transcriptome data of the currently sampled growth period. Given the above, the current genomic and transcriptomic data provides valuable insights into the evolution and metabolites analysis of *S*. *sanghuang*.

## 1. Introduction

*Sanghuangporus*, belonging to *Basidiomycota*, *Agaricomycetes*, *Hymenochaetales*, *Hymenochaetaceae*, is a genus of wood-inhabiting macrofungus with important medicinal values [1,2]. The taxonomic position of 18 species in this genus has been confirmed, and ten of them are widely distributed in China [3,4,5,6]. Owing to its significant medicinal properties, *Sanghuangporus* has mainly been utilized in the form of tea in China and adjacent countries for more than 2000 years [2,7]. Modern scientific studies indicated that *Sanghuangporus* is a rich source of bioactive secondary metabolites, comprising polysaccharides, flavonoids, phenols, terpenes, steroids, coumarins, alkaloids, and others [7]. The various pharmacological activities of these compounds, such as antioxidant properties [8], lowering blood glucose [9], immune regulation [9], and antibacterial and anti-inflammatory activities [10], have also been confirmed, thus leading to *Sanghuangporus* becoming a hotspot in scientific research and commercial applications [11,12].

High-throughput sequencing technology provides an unprecedented sequencing capacity that enables whole-genome and transcriptome sequencing to be completed at a low cost and in a short period, and thus facilitates omics mining and the transformation of fungal secondary metabolite biosynthesis genes [13]. Over the past decades, the genomes and transcriptomes of various fungi have been sequenced and analyzed, which improves the understanding of fungal growth and development. Unfortunately, for wood-inhabiting macrofungi, especially those in *Hymenochaetaceae*, information on genome and transcriptome sequences is largely lacking. Up to now, the genome sequences of only 15 species in *Hymenochaetaceae* have been generated and released in the NCBI database. Of these 15 species, 3 are from *Sanghuangporus*, including the generic type *S*. *sanghuang*, being considered to be the most precious medicinal fungus in this genus [2,7]. Although the genome of *S*. *sanghuang* itself was sequenced well from the monokaryon strain MS2 by our lab [14], its assembly and annotation still can be improved. A widely accepted view is that highly accurate and comprehensive genome assembly and annotation are prerequisites for genome mining and functional genomics research [15]. Therefore, the continuous revision of the genome annotation of *S*. *sanghuang* is an important foundation for the further utilization of this fungal resource.

Moreover, supplementing transcriptomic data is a crucial approach for improving the accuracy of genome annotation, and a well-annotated genome also can facilitate the transcriptomic analyses of genes involved in the biosynthesis of secondary metabolites. Indeed, genomic data in association with transcriptome analyses have been of considerable benefit to the medicinal studies of some wood-inhabiting fungi, such as *Ganoderma lucidum* [16], *Taiwanofungus camphoratus* [17], and *Hericium erinaceus* [18]. For example, by sequencing and analyzing the genome and transcriptome of *G*. *lucidum*, it was found that the differentiation of secondary metabolite synthesis gene clusters was usually accompanied by the generation of new medicinal activities [16]. In *T*. *camphoratus*, the genes involved in terpene synthesis reached the highest expression level at the fruiting body stage [19]. Regarding *Sanghuangporus*, the differential expression of genes involved in synthesizing secondary medicinal metabolites at different growth stages have been preliminarily revealed from *Sanghuangporus vaninii* with the help of genomic and transcriptomic data [20,21]. However, the transcriptomic data of *S*. *sanghuang* is still unreported, which limits the accurate recognition of genes and gene clusters related to medicinal properties.

In this study, the originally annotated genome of the *S*. *sanghuang* strain MS2 is updated by supplementing the transcriptomic data newly sequenced from the same strain. Then, the original genome annotation, named MS2 version 1 annotation (MS2_V1), the currently updated genome annotation, named MS2 version 2 annotation (MS2_V2), and the transcriptome annotation are compared, particularly in terms of the genes and gene clusters related to medicinal applications. The updated genome and transcriptome data in this study could provide a reliable basis for the further application of *S*. *sanghuang*.

## 2. Materials and Methods

### 2.1. Biological Material

The monokaryotic strain MS2 of *S*. *sanghuang* used in our previous paper [14] was preserved under standard conditions. After incubation, the strain MS2 was transferred to a potato dextrose agar (PDA) plate for ten days’ cultivation at 28 °C in the dark. After that, the mycelia grew over the whole PDA plate and were then harvested for transcriptome sequencing.

### 2.2. Transcriptome Sequencing

The mycelia of *S*. *sanghuang* strain MS2 were sent to Personalbio (Nanjing, China) for RNA extraction, cDNA library construction, and sequencing. The total RNA was extracted and converted into a cDNA library using an Illumina TruSeq RNA sample preparation kit with oligo(dT) magnetic beads. Paired-end (PE) sequencing of cDNA libraries was performed based on the HiSeq sequencing platform. Primers and reads with an average quality score less than Q20 were removed from the primary sequencing data (raw reads) using cutadapt [22]. The output clean reads were subjected to subsequent analyses.

### 2.3. Genome Assembly and Prediction

**Figure 1:**
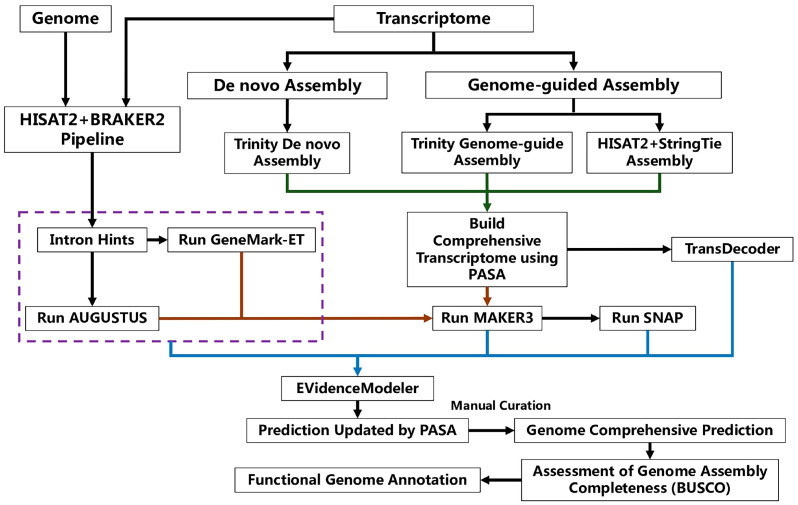
Schematic illustration of pipeline for the genome re-annotation of *Sanghuangporus sanghuang* strain MS2 with the supplements of transcriptome sequences. The purple dotted box indicates the training of gene models using the BRAKER2 pipeline. The green lines indicate the import of three assemblies in PASA for building a more comprehensive transcriptome database. The brown lines indicate the import of transcripts in MAKER3 for the further prediction of gene models. The blue lines indicate the integration of all transcriptome databases using EVidenceModeler.

The original genome of *S*. *sanghuang* strain MS2 were subjected to an updated annotation with the help of RNA-seq data following the pipeline in Figure 1.

First, RNA clean reads were mapped to the repetitive sequence-masked genome of *S*. *sanghuang* MS2 using BUSCO (Benchmarking Universal Single-Copy Orthologs) v2.2.1 [23]. The mapped reads were further sorted and indexed using SAMtools v1.16.1 [24]. The unmasked genome and the mapped reads were then input into BRAKER2 v2.1.6 [25] to do ab initio prediction using default settings. After identifying the introns, preliminary training datasets for the prediction of gene models were generated using GeneMark-ET v4.46 and AUGUSTUS v3.4.0, both implemented in BRAKER2 v2.1.6.

Then, the combined strategies of de novo assembly and genome-guided assembly were utilized. The de novo transcriptome assembly was performed using Trinity v2.8.5 [26] with the option jaccard_clip under the standard pattern. Regarding the genome-guided assembly, transcripts were assembled and merged using Trinity v2.8.5 and StringTie v2.2.1 [27], and HISAT2 v2.2.1 was used with default settings for the reads mapped above. These three assemblies were combined to obtain a more comprehensive transcriptome database using PASA (Program to Assembly Spliced Alignments) v2.5.2 [28]. On one side, the resulting comprehensive transcriptome database was subjected to the prediction of gene models using TransDecoder v5.5.0 [26]. On the other side, the resulting comprehensive transcriptome database, genome sequences of *S*. *sanghuang* strain MS2, repetitive sequences previously identified from *S*. *sanghuang* strain MS2 [14], and all available fungal homologous protein sequences in the UniProtKB/Swiss-Prot Protein Sequence Database (https://www.uniprot.org/, accessed on 13 September 2022) were first subjected to generate an initial prediction of the gene model using the SNAP model implemented in MAKER3 v3.01.03 [29]. Then, this initial prediction, the comprehensive transcriptome database, and the preliminary training datasets generated by BRAKER2 v2.1.6 were incorporated into MAKER3 v3.01.03 for the further prediction of the gene models.

Finally, the gene models predicted from BRAKER2 v2.1.6, TransDecoder v5.5.0, MAKER3, and the comprehensive transcriptome database were integrated using EVidenceModeler v1.1.1 [30] with weights of 8, 9, 6, and 10, respectively. The resulting gene models were updated by PASA and a careful manual curation to obtain the final prediction of the gene models.

The updated genome assembly was submitted to the National Microbiology Data Center (NMDC, https://nmdc.cn/, accessed on 5 January 2023) with accession number NMDC60046375.

### 2.4. Transcriptome Assembly

The RNA clean reads were mapped to the updated assembly of the reference genome using HISAT2 v2.2.1 [23], and further sorted and indexed using SAMtools v1.16.1 [24]. Moreover, the alignments were assembled using StringTie v2.2.1 [27] from the indexed mapped reads with default settings. Eventually, the transcripts were converted into open reading frames (ORFs) and proteins using TransDecoder v5.5.0 [26]. The transcriptome assembly was submitted to NMDC with accession number NMDC60046376.

### 2.5. Gene Annotation

To ensure the comparability of the gene functions, the original genome, the updated genome, and the transcriptome of *S*. *sanghuang* strain MS2 were annotated simultaneously. The following databases were selected as references: the Non-Redundant Protein Database (NR) (https://www.ncbi.nlm.nih.gov/protein/, accessed on 13 September 2022), UniProtKB/Swiss-Prot Protein Sequence Database (https://www.uniprot.org/, accessed on 13 September 2022), KOG (Eukaryotic Orthologous Groups, https://www.creative-proteomics.com/services/kog-annotation-analysis-service.htm, accessed on 13 September 2022), InterProScan (http://www.ebi.ac.uk/InterProScan/, accessed on 13 September 2022), Pfam (http://pfam.xfam.org/, accessed on 27 February 2020), Fungal Transcription Factor Database (FTFD, http://ftfd.snu.ac.kr/, accessed on 13 September 2022), Carbohydrate-Active enZYmes (CAZymes) Database (http://www.cazy.org/, accessed on 13 September 2022), eggNOG-mapper (http://eggnog-mapper.embl.de/, default parameters for “Auto adjust per query”, accessed on 13 September 2022), Kyoto Encyclopedia of Genes and Genomes (KEGG, https://www.kegg.jp/, accessed on 13 September 2022), Gene Ontology (http://geneontology.org/, accessed on 13 September 2022), and Fungal Cytochrome P450 Database (http://p450.riceblast.snu.ac.kr/cyp.php, accessed on 13 September 2022). All predicted coding genes were aligned with these databases using DIAMOND v2.0.2 [31] with the cut-off values of E-value no more than 1 × 10^−5^, identity not less than 40%, and coverage not less than 40%. The completeness of the genome annotations was assessed using BUSCO v5.2.2 [32] (database: basidiomycota_odb10, accessed on 11 July 2022).

Furthermore, biosynthetic gene clusters encoding potential secondary metabolites were identified using AntiSMASH (https://fungismash.secondarymetabolites.org/, accessed on 13 September 2022).

The annotation was submitted to NMDC with accession number NMDCX0000165.

### 2.6. Phylogenetic Analysis of Cytochrome P450 (CYP)

The full-length protein sequences of CYPs were first aligned using MUSCLE implemented in MEGA7 with default settings, and then a phylogenetic tree was constructed using the Neighbor-Joining method [33]. The NCBI CDD (Conserved Domain Database, https://www.ncbi.nlm.nih.gov/cdd/, accessed on 13 September 2022) was used to screen the conserved domains of CYPs, which were subjected to the prediction of motifs using the Simple MEME program implemented in TBtools [34]. The visualization of the phylogenetic tree, motif, and conserved domains with their classifications and gene structures were conducted using the Gene Structure View tool implemented in TBtools.

## 3. Results

### 3.1. Transcriptome Sequencing and Updated Genome Assembly

**Figure 2:**
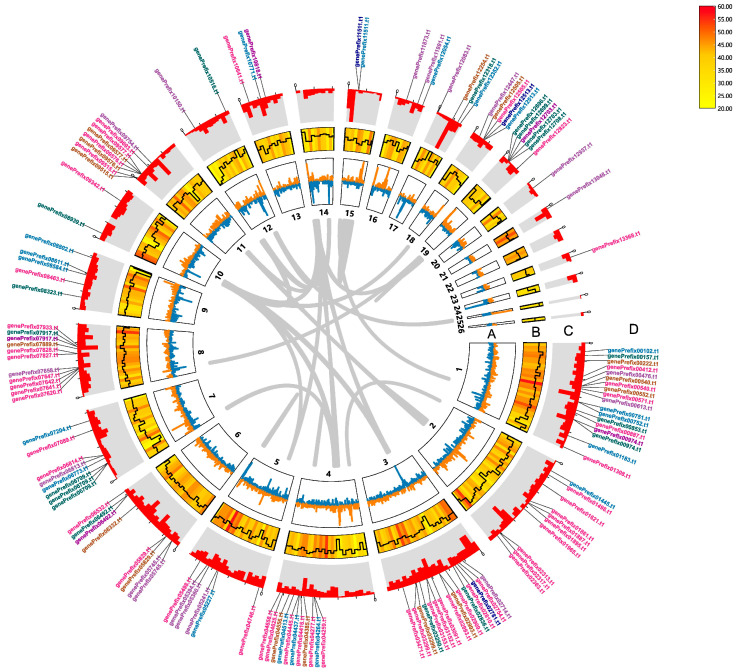
Characteristics of re-annotated genome of *Sanghuangporus sanghuang* strain MS2. From outside to inside are as follows: (**A**) contigs and GC skew: calculated as the percentage of (G − C)/(G + C) in 1 kb non-overlapping windows, and the inward blue part represents G/C < 1, while the outer orange part represents G/C > 1; (**B**) gene density: indicated by the heat map in red and yellow colors with a solid black line representing GC ratio; (**C**) transcriptome sequences mapped to the genome; (**D**) position of transcripts related to the biosynthesis of secondary metabolites in the genome. Lavender, blue, pink, brown, green, azure, and purple characters, respectively, represent genes involved in terpenoid backbone biosynthesis, sesquiterpenoid and triterpenoid biosynthesis, polysaccharide biosynthesis, uridine diphosphate glucose biosynthesis, ubiquinone and other terpenoid-quinone biosynthesis, steroid biosynthesis, and phenylpropanoid biosynthesis.

A total of 43,147,698 raw reads composed of 6.47 Gb sequences were generated from *S*. *sanghuang* strain MS2 via paired-end transcriptome sequencing using an Illumina HiSeq 2000. The Q30 and Q20 percentages were 94.24% and 97.99%, respectively, indicating the low sequencing error rate. After filtering the low-quality sequences, a total of 40,515,552 clean reads comprising 6,077,332,800 sequences remained (Supplementary Table S1). From these clean reads, de novo assembly and genome-guided assembly generated a total of 683,416 transcripts. On the basis of these transcripts, the prediction of genome sequences from *S*. *sanghuang* strain MS2 were updated (Table 1) and the 26 contigs were visualized using Circos implemented in TBtools (Figure 2).

### 3.2. Transcriptome Assembly

With the updated genome of *S*. *sanghuang* strain MS2 as a reference, the clean reads were assembled to 13,531 transcripts with an average length of 1266.30 bp, an N50 length of 1698 bp, and a GC content of 51% (Supplementary Table S1).

### 3.3. Comparison of MS2_V1 and MS2_V2 Assemblies

The MS2_V2 assembly of *S*. *sanghuang* strain MS2 contains 13,531 protein-coding genes with an average gene length of 1582.25 bp, of which 2618 genes are newly predicted compared to the MS2_V1 assembly (Table 1). Besides the number of protein-coding genes, the total length of the protein-coding genes in MS2_V2 (21.41 Mb) is also higher than that in MS2_V1 (21.05 Mb, Table 1). Moreover, the functional annotation generated from MS2_V2 assembly has a complete BUSCOs of 92.8%, significantly increasing than that from MS2_V1 assembly (11.7%, Table 1). All of these data indicate a remarkable improvement in the quality of genome assembly.

### 3.4. Functional Annotation of Protein-Coding Genes

Among the various databases, the highest number of genes in MS2_V2 was annotated from eggNOG followed by InterProScan, Pfam, GO, NR, Swiss-Prot, KEGG, KOG, CAZymes, and FTFD (Table 2). This trend is the same for MS2_V1 and transcriptome annotation (Table 2). Compared to MS2_V1, the gene numbers annotated in MS2_V2 are higher than all of the above databases (Table 2), suggesting that the re-annotation by supplementing the transcriptomic data improves the functional recognition of the *S*. *sanghuang* strain MS2 genome.

**Figure 3:**
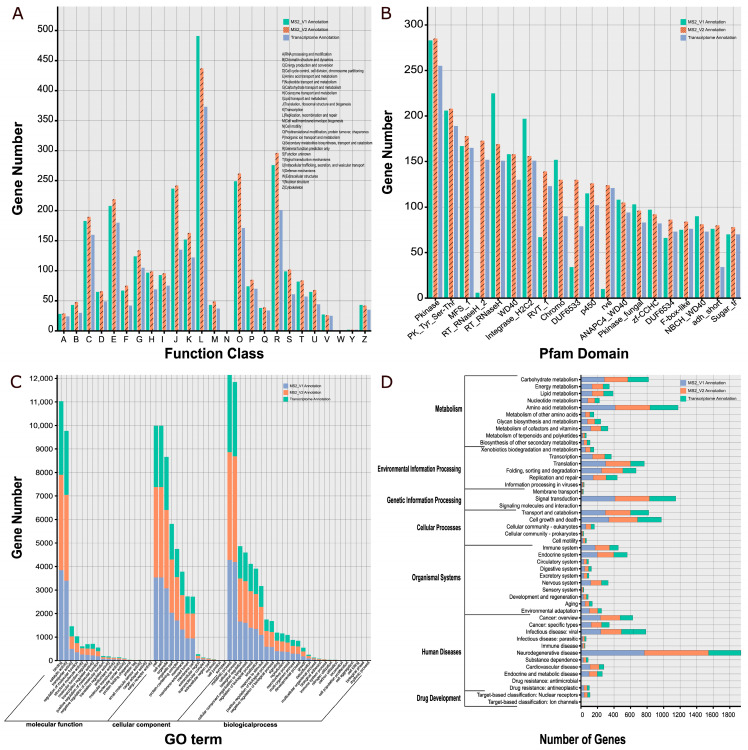
Comparison of gene functions in the original genome annotation (MS2_V1), the currently updated genome annotation (MS2_V2), and the transcriptome annotation of *Sanghuangporus sanghuang* strain MS2. (**A**) COG annotation, (**B**) Pfam domain, (**C**) GO annotation, (**D**) KEGG annotation.

All functional categories, except extracellular structures, were annotated in MS2_V1, MS2_V2, and the transcriptome from the KOG database (Figure 3A). For MS2_V2, the mostly enriched functional categories include replication, recombination and repair (437 genes), general functional prediction only (296 genes), post-translational modifications, protein turnover, chaperones (262 genes) and translation, ribosome structure, and biogenesis (242 genes). Of these categories, a higher number of genes were annotated in MS2_V2 compared to MS2_V1, except the category of replication, recombination, and repair. Moreover, the transcriptome annotation shows substantial numbers of genes involved in replication, recombination, and repair (373 genes), general functional predictions (201 genes), and amino acid transport and metabolism (180 genes).

Three Pfam domain genes of highest abundances are Pkinase (253 genes in the former and 255 genes in the latter), PK_Tyr_Ser-Thr (208 genes in the former and 189 genes in the latter) and MFS_1 (178 genes in the former and 165 genes in the latter) both in MS2_V2 and transcriptome annotation (Figure 3B).

A total of 59 GO terms were annotated in MS2_V2 and transcriptome annotation from gene ontology, viz. biological process (20,534 genes in the former and 14,975 genes in the latter), cellular component (18,910 genes in the former and 12,804 genes in the latter), and molecular function (9939 genes in the former and 7449 genes in the latter) (Figure 3C). Of these GO terms, the largest number of genes is involved in metabolic processes, followed by cellular processes, binding, cell, cell part, catalytic activity, and organelles (Figure 3C).

Regarding the KEGG database, most annotated genes are involved in the functions of neurodegenerative disease (776 genes in MS2_V2 and 391 genes in transcriptome annotation), amino acid metabolism (421 genes in MS2_V2 and 340 genes in transcriptome annotation), signal transduction (417 genes in MS2_V2 and 318 genes in transcriptome annotation), and cell growth and death (346 genes in MS2_V2 and 289 genes in transcriptome annotation) (Figure 3D).

### 3.5. Identification of Genes Involved in Synthesis of Secondary Metabolites

#### 3.5.1. Terpenoid Biosynthesis

Terpenoids are one of the primary and secondary metabolites in *S*. *sanghuang*. A total of 17 key enzymes encoded by 18 genes involved in terpenoid backbone biosynthesis were identified from MS2_V2, two more genes than those in MS2_V1 (Supplementary Figures S1 and S2, Table S2). Regarding transcriptome annotation, 13 key enzymes encoded by 14 genes were identified, seven of which were via the mevalonate (MVA) pathway (Supplementary Figure S3, Table S2). All of these key enzymes are encoded by single- or double-copy genes (Supplementary Table S2). In addition, the same three genes involved in sesquiterpene and triterpenoid biosynthesis that is indirectly related to terpenoid biosynthesis were identified from MS2_V1, MS2_V2, and transcriptome annotation (Supplementary Figure S4, Table S3).

#### 3.5.2. Polysaccharide Biosynthesis

In this study, we identified 24 genes encoding polysaccharide biosynthesis (starch and sucrose metabolism) in MS2_V2, one less than MS2_V1 (Supplementary Figures S5 and S6, Table S4). Most of these enzymes are encoded by single-, double-, and triple-copy genes, while the endoglucanase, the beta-glucosidase, and the glucan 1,3-beta-glucosidase are encoded by four-, seven-, and nine-copy genes, respectively (Supplementary Table S4). Of these, 15 key enzymes encoded by 35 genes were identified in transcriptome annotation, indicating that the polysaccharide biosynthesis of the strain MS2 is active during this growth period (Supplementary Figure S7, Table S4). In addition, 11 enzymes encoded by 15 genes were identified from MS2_V2 to be involved in the biosynthesis of uridine diphosphate glucose, the precursor of glucans, one more gene than those from MS2 _V1 (Supplementary Table S5).

#### 3.5.3. Ubiquinone and Other Terpenoid Quinone Biosynthesis

In MS2_V2, 9 enzymes encoded by 18 genes involved in the biosynthesis of ubiquinone and other terpenoid quinones were annotated, which is 6 more genes than those in MS2_V1 (Supplementary Figures S8 and S9, Table S6). Nevertheless, only 7 key enzymes encoded by 12 genes were identified in transcriptome annotation (Supplementary Figure S10, Table S6).

#### 3.5.4. Steroid Biosynthesis

Comparatively, 15 enzymes encoded by 19 genes involved in the biosynthesis of steroids were identified from MS2_V2, 1 more enzyme and 1 more gene than those in MS2_V1 (Supplementary Figures S11 and S12, Table S7), and 2 more enzymes and 3 more genes than those in transcriptome annotation (Supplementary Figure S13, Table S7).

#### 3.5.5. Flavonoid Biosynthesis

As in previous studies, the MS2_V2 of *S*. *sanghuang* was not annotated with any essential enzymes directly related to the pathway for the flavonoid biosynthesis, flavone and flavonol biosynthesis, and anthocyanin biosynthesis. However, as an upstream process of flavonoid biosynthesis, the phenylpropanoid biosynthetic pathway was shown to encode two enzymes through five genes (Supplementary Figure S14, Table S8). Moreover, the same results were identified in transcriptome annotation (Supplementary Figure S14, Table S8).

### 3.6. CAZyme

For CAZyme profiles, a total of 447 genes were identified in MS2_V2, including 30 carbohydrate-binding modules (CBMs), 49 carbohydrate esterases (CEs), 193 glycoside hydrolases (GHs), 57 glycosyltransferases (GTs), 29 to polysaccharide lyases (PLs) and 89 auxiliary activities (AA) (Table 3). In addition, 344 genes encoding CAZymes were identified in transcriptome annotation (Table 3), with the families GH16 (23 genes), GH5 (23 genes), AA7 (13 genes), AA (12 genes), and CBM1 (10 genes) encoded by 10 or more genes.

### 3.7. CYP

It has been demonstrated that some CYP genes in medicinal fungi can be involved in the synthesis of terpenoids and sterols [35]. A total of 127 CYP genes were screened in MS2_V2, 8 more than those in MS2_V1, while 103 CYP genes were found in the transcriptome annotation (Table 3). Among these genes, the largest number was identified from E-class P450, group I (75 genes in MS2_V2 and 64 genes in transcriptome annotation), followed by P450, CYP52 (7 genes in MS2_V2 and 7 genes in transcriptome annotation), E-class P450, group IV (7 genes in MS2_V2 and 4 genes in transcriptome annotation), and Pisatin demethylase-like (6 genes in MS2_V2 and 5 genes in transcriptome annotation) (Table 3). Moreover, 32 and 23 CYP genes, respectively, in MS2_V2 and transcriptome annotation cannot be accurately identified in any known class (Table 3).

**Figure 4:**
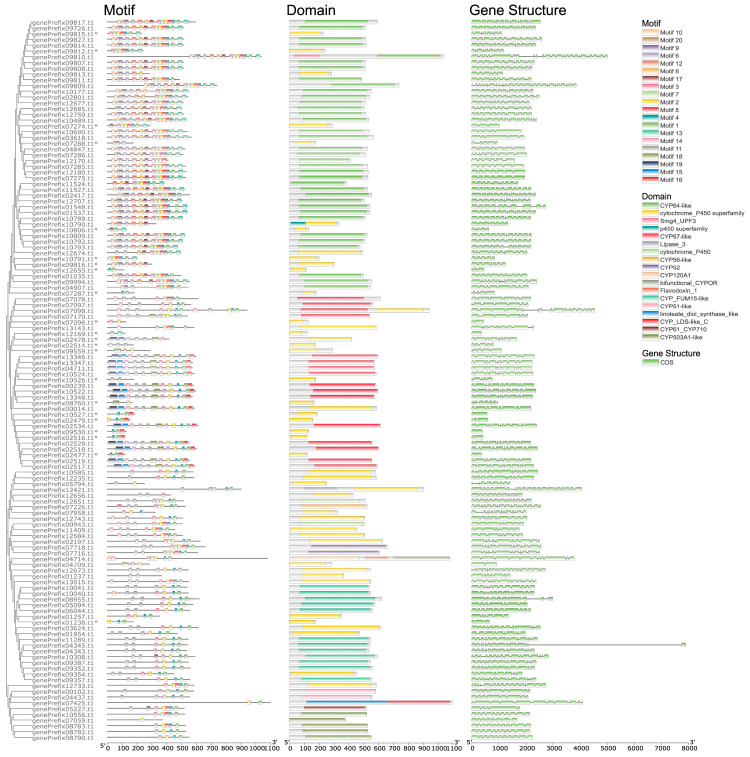
Phylogenetic relationships of 125 genes encoding Cytochromes P450 from the re-annotated genome of *Sanghuangporus sanghuang* strain MS2, and their predicted motifs, domains, and gene structures. * indicates that the gene is not found in the transcriptome annotation.

The above-identified CYP genes were finally grouped into 18 classes (Supplementary Tables S9–S11). Most of these genes have ten or more motifs and each gene has one to three domains, which are visualized together with their gene structures in a phylogenetic tree of CYP genes after removing two genes that lack common sites with others and contain only two motifs (Figure 4). Noteworthily, 17.6% of these CYP genes cannot be found in the current transcriptome annotation (Figure 4).

### 3.8. Gene Cluster of Secondary Metabolites

From MS2_V2, twenty gene clusters were predicted, of which four were iterative type I polyketide synthases (T1PKS), five were non-ribosomal peptide-like synthases (NRPS-like) and eleven were found to encode terpenoids (Table 3). In contrast to MS2_V1, no exact gene cluster encoding a non-ribosomal peptide synthase (NRPS) was found (Table 3).

## 4. Discussion

### 4.1. Methodology of Genome Re-Annotation

Genome annotation is a high-throughput annotation of the biological functions of all genes in the genome using bioinformatic methods and tools, which is a hot topic in functional genomics. The current study aims to improve the utilization of the *S*. *sanghuang* genomic resource, thereby enhancing our understanding of the biology of the macrofungi.

To date, the genome prediction and annotation methods used for species in *Basidiomycota* are quite different. Some fungal genomes were annotated using only one de novo annotation software program. For instance, the *Inonotus obliquus* genome was annotated with BRAKER [36] and the *Russula griseocarnosa* genome with MAKER [37]. Sometimes, multiple programs are simultaneously performed, such as the genome of *Ganoderma leucocontextum* annotated using six software packages [38]. In addition, directly using the JGI Annotation Pipeline is another option [39,40]. However, many of the previous genome annotations still employ the ab initio prediction model, which is prone to result in false positives, false negatives, unpredictable UTR regions, and the inaccurate identification of alternative splicing sites. Indeed, the utilization of more genome structure prediction software programs will lead to more false positives. Therefore, the strategy of combining BRAKER2 and MAKER3 is used in the current study, which is of considerable benefit to the efficiency of genome prediction.

Besides bioinformatic algorithms, new data are also important to improve the accuracy of genome annotation. Among two dozen genome-sequenced species in *Hymenochaetales*, only *Phellinus noxius* was annotated with the reference of both corresponding transcriptome data and protein sequences of *Hymenochaetales* available from the UniProtKB/Swiss-Prot database [41]. These data are helpful for reducing inaccurate predictions. Accordingly, the transcriptome data of *S*. *sanghuang* strain MS2 and all available fungal homologous protein sequences from the UniProtKB/Swiss-Prot database were utilized to supplement the genome prediction of *S*. *sanghuang* strain MS2.

Currently, the updated genome assembly of the *S*. *sanghuang* strain MS2 has been significantly improved in terms of the accuracy and completeness of gene models, most obviously an increase of 2618 predicted protein-coding genes and 81.1% more BUSCOs completeness in the optimized data compared to the original data [14]. A clearer picture of the gene structure, copy number, and transcripts of *S*. *sanghuang* is achieved, further facilitating the genome annotation. Moreover, the functional categories and numbers of protein-coding genes annotated in MS2_V2 according to 10 databases have been polished compared with MS2_V1. In addition, more functional genes related to medicinal component synthesis were identified from the updated genome, such as in terpenoid backbone biosynthesis, ubiquinone and other terpenoid quinone biosynthesis, and uridine diphosphate glucose biosynthesis. In addition, a large number of genes related to secondary metabolism were identified in the transcriptome of *S*. *sanghuang* strain MS2, and the accurate prediction of these genes could play an important role in guiding the subsequent multidisciplinary analysis of genetics and pharmacology. Nevertheless, the current transcriptome data were sequenced from only one growth period. It is expected that more comprehensive transcriptome data from various growth periods will further improve the genome annotation of *S*. *sanghuang* strain MS2.

### 4.2. Substantial Transcription of Genes Related to Secondary Metabolite Biosynthesis

In Asia, *S*. *sanghuang* is widely used for its ability to produce hundreds of secondary metabolites [7]. Among the various parameters that determine the expression level of a gene, transcription initiation is the first critical step and the most regulated step in gene expression in all organisms [42]. Compared with genome data, transcripts can provide a more direct clue to the production of medicinal secondary metabolites and play an important role in guiding the metabolite analysis of gene expression. In the sampled growth period, a large number of transcripts related to the biosynthesis of secondary metabolites were identified from 21 contigs of the genome of *S*. *sanghuang* strain MS2 (Figure 2). It was revealed that *S*. *sanghuang*, in the current condition, could accurately produce a relatively large quantity of transcripts related to active substance synthesis pathway, such as polysaccharide biosynthesis, steroid biosynthesis, and terpene skeleton biosynthesis (Table 3). In particular, the number of genes involved in the polysaccharide biosynthesis in transcriptome annotation is 35 (Table 3), which is almost two times more than that in the previously reported transcriptomes of *S*. *vaninii* (19 genes) [21]. Specifically, only the genes associated with glucan synthesis were annotated in the *S*. *vaninii* transcriptome, while some genes related to the synthesis of cellulose and trehalose were additionally annotated in the *S*. *sanghuang* transcriptome annotation. That is to say that *S*. *sanghuang*, in the sampled growth period, at least possesses a strong ability for polysaccharide synthesis, which needs to be further confirmed by comparative transcriptome analysis. Regarding CYPs, they have been demonstrated to be vital in synthesizing fungal secondary metabolites, mainly concentrating on the biosynthetic metabolic processes of terpenoids and sterols [43]. In the current case, 127 CYP genes were annotated in MS2_V2, 8 more than in MS2_V1, while 103 CYP genes were identified in the transcriptome annotation (Table 3). Considering the sequence characteristics of motifs binding as transcription factor binding sites, the binding sites of transcription factors can be clarified, which will help reveal the biological functions and mechanisms of these transcription factors. Differences in the number and arrangement of motifs of the 25 untranscribed CYP genes could be observed (Figure 4). Combined with previous reports, we speculate that the number of these motifs in CYP genes may contribute to the above result [44].

Consequently, the currently sampled growth period is suitable for the extraction, isolation, and purification of medicinal compounds such as polysaccharides, steroids, terpenes, uridine diphosphate glucose, and ubiquinone and other terpene quinones. In contrast, regarding obtaining sesquiterpenoids, triterpenoids, and certain other medicinal secondary metabolites, more suitable growth periods still need to be determined.

### 4.3. Identification of Genes Related to Flavonoid Synthesis

*S*. *sanghuang* is known to have the ability to produce a variety of flavonoids with antioxidant, anti-proliferative, and anti-microbial activities [45]. Compared with *S*. *vaninii*, the genes related to flavonoid synthesis in *S*. *sanghuang* only identified the upstream pathway involved in the biosynthesis of flavonoids, viz. the phenylpropanoid biosynthesis pathway, and there were no key enzymes directly related to flavonoid biosynthesis, flavonoid and flavonol biosynthesis, and anthocyanin biosynthesis pathways in the MS2_V1, MS2_V2, and transcriptome annotation of *S*. *sanghuang* strain MS2 [21]. It is tempting to speculate that the absence of key enzymes directly related to the flavonoid biosynthesis pathways in *S*. *sanghuang* strain MS2 may be due to the interspecific difference, and the absence of relevant omics data in various databases may also play a part. Above all, the specific pathways for flavonoid biosynthesis in *Sanghuangporus* are still in the process of exploration, and it is essential to conduct comparative studies combining different species of different genera and different specimens of the same species to obtain the specific biosynthesis mode.

## 5. Conclusions

In summary, the current re-annotated genome of *S*. *sanghuang* strain MS2 has shown a significant improvement in terms of accuracy and completeness. More functional genes related to medicinal applications are revealed from the updated genome than the original one. Moreover, a substantial percentage of genes involved in the biosynthesis of medicinal secondary metabolites are identified during the sampled growth period of *S*. *sanghuang* strain MS2, making them suitable for subsequent multidisciplinary analyses, including genetics and pharmacology. Besides facilitating the application of *S*. *sanghuang* itself, the current bioinformatic pipeline, especially integrating transcriptome data and all available fungal homologous protein sequences in the UniProtKB/Swiss-Prot Protein Sequence Database, is also an important reference for genome assembly and the annotation of other fungi.

## Figures and Tables

**Table 1:** Information of genome assemblies of *S*. *sanghuang* strain MS2 in the original genome annotation (MS2_V1) and the currently updated genome annotation (MS2_V2).

Item	MS2_V1	MS2_V2
Number of protein-coding genes	10,913	13,531
Total length of protein-coding genes (bp)	21,053,817	21,409,391
Average length of protein-coding genes (bp)	1929.24	1582.25
Average exon length (bp)	267.00	236.43
Average CDS length (bp)	1559.82	1266.30
Complete BUSCOs (C)	11.7% (207)	92.8% (1637)
Complete and single-copy BUSCOs (S)	11.7% (207)	92.5% (1632)
Complete and duplicated BUSCOs (D)	0% (0)	0.3% (5)
Total BUSCO groups searched	1764	1764

**Table 2:** Comparison of annotated gene numbers from various databases in the original genome annotation (MS2_V1), the currently updated genome annotation (MS2_V2), and the transcriptome annotation of *Sanghuangporus sanghuang* strain MS2.

Database	MS2_V1	MS2_V2	Transcriptome
NR	6033 (55.38%)	6404 (47.33%)	4480 (33.11%)
Swiss-Prot	5378 (49.28%)	5681 (41.99%)	4103 (30.32%)
KOG	2381 (21.82%)	2444 (18.06%)	1770 (13.08%)
eggNOG	8362 (76.63%)	9215 (68.10%)	6140 (45.38%)
Interproscan	8055 (73.83%)	8828 (65.24%)	6083 (44.96%)
Pfam	7968 (73.01%)	7968 (58.89%)	5583 (41.26%)
GO	6387 (58.53%)	6910 (51.07%)	4939 (36.50%)
KEGG	3743 (34.30%)	3972 (29.35%)	2840 (20.99%)
CAZymes	401 (3.67%)	448 (3.31%)	335 (2.48%)
FTFD	321 (2.94%)	358 (2.65%)	296 (2.19%)
Total number	10,913	13,531	13,531

**Table 3:** Comparison of certain functional genes in the original genome annotation (MS2_V1), the currently updated genome annotation (MS2_V2), and the transcriptome annotation of *Sanghuangporus sanghuang* strain MS2.

Characteristic	Item	MS2_V1	MS2_V2	Transcriptome
Gene involved in the pathway of medicinal metabolites	Terpenoid backbone biosynthesis	16	18	14
	Ubiquinone and other terpenoid-quinone biosynthesis	13	17	12
	Sesquiterpenoid and triterpenoid biosynthesis	3	3	3
	Polysaccharide biosynthesis	53	52	35
	Steroid biosynthesis	19	19	16
	Uridine diphosphate glucose biosynthesis	14	15	14
	Phenylpropanoid biosynthesis	5	5	5
Gene encoding CAZymes	CBM	22	30	19
	CE	30	49	21
	GH	187	193	156
	GT	55	57	56
	PL	27	29	21
	AA	79	89	61
	Sum	400	447	334
Gene encoding cytochromes P450	Pisatin demethylase-like	7	6	5
	P450, CYP52	8	7	7
	E-class P450, group IV	6	7	4
	E-class P450, group I	71	75	64
	Undetermined	21	32	23
	Sum	119	127	103
Gene cluster of secondary metabolites	terpene	12	11	--
	T1PKS	4	4	--
	NRPS	1	0	--
	NRPS-like	4	5	--
	Sum	21	20	--

## Data Availability

Publicly available datasets were analyzed in this study. The updated genome and transcriptome assemblies were deposited in the China National Microbiology Data Center (NMDC; https://nmdc.cn/, accessed on 5 January 2023) with accession numbers NMDC60046375 and NMDC60046376, respectively. The detailed annotation data of genomes and transcriptomes were deposited in the China National Microbiology Data Center (NMDC) with accession number NMDCX0000165.

## Supplementary Materials

The following are available online at https://www.mdpi.com/article/10.3390/jof9050505/s1. Figure S1: Pathway of terpenoid backbone biosynthesis in MS2_V1 of *Sanghuangporus sanghuang* strain MS2. The blue and green boxes indicate the presence of single- and double-copy homologous genes, respectively. Figure S2: Pathway of terpenoid backbone biosynthesis in MS2_V2 of *Sanghuangporus sanghuang* strain MS2. The blue and green boxes indicate the presence of single- and double-copy homologous genes, respectively. Figure S3: Pathway of terpenoid backbone biosynthesis in transcriptome annotation of *Sanghuangporus sanghuang* strain MS2. The blue and green boxes indicate the presence of single- and double-copy homologous genes, respectively. Figure S4: Pathway of sesquiterpenoid and triterpenoid biosynthesis in MS2_V1, MS2_V2, and transcriptome annotation of *Sanghuangporus sanghuang* strain MS2. The blue boxes indicate the presence of single-copy homologous genes. Figure S5: Pathway of polysaccharide biosynthesis (starch and sucrose metabolism) in MS2_V1 of *Sanghuangporus sanghuang* strain MS2. The blue, green, orange, yellow, gray, purple, and pink boxes indicate the presence of single-, double-, triple-, four-, five-, seven-, and ten-copy homologous genes, respectively. Figure S6: Pathway of polysaccharide biosynthesis (starch and sucrose metabolism) in MS2_V2 of *Sanghuangporus sanghuang* strain MS2. The blue, green, orange, yellow, purple, and red boxes indicate the presence of single-, double-, triple-, four-, seven-, and nine-copy homologous genes, respectively. Figure S7: Pathway of polysaccharide biosynthesis (starch and sucrose metabolism) in transcriptome annotation of *Sanghuangporus sanghuang* strain MS2. The blue, green, orange, yellow, and red boxes indicate the presence of single-, double-, triple-, four-, and nine-copy homologous genes, respectively. Figure S8: Pathway of ubiquinone and other terpenoid quinone biosynthesis in MS2_V1 of *Sanghuangporus sanghuang* strain MS2. The blue, green, and yellow boxes indicate the presence of single-, double-, and four-copy homologous genes, respectively. Figure S9: Pathway of ubiquinone and other terpenoid quinone biosynthesis in MS2_V2 of *Sanghuangporus sanghuang* strain MS2. The blue, green, orange, and yellow boxes indicate the presence of single-, double-, triple-, and four-copy homologous genes, respectively. Figure S10: Pathway of ubiquinone and other terpenoid quinone biosynthesis in transcriptome annotation of *Sanghuangporus sanghuang* strain MS2. The blue, green, and yellow boxes indicate the presence of single-, double-, and four-copy homologous genes, respectively. Figure S11: Pathway of steroid biosynthesis in MS2_V1 of *Sanghuangporus sanghuang* strain MS2. The blue and green boxes indicate the presence of single- and double-copy homologous genes, respectively. Figure S12: Pathway of steroid biosynthesis in MS2_V2 of *Sanghuangporus sanghuang* strain MS2. The blue and green boxes indicate the presence of single- and double-copy homologous genes, respectively. Figure S13: Pathway of steroid biosynthesis in transcriptome annotation of *Sanghuangporus sanghuang* strain MS2. The blue and green boxes indicate the presence of single- and double-copy homologous genes, respectively. Figure S14: Pathway of phenylpropanoid biosynthesis in MS2_V1, MS2_V2, and transcriptome annotation of *Sanghuangporus sanghuang* strain MS2. The blue and yellow boxes indicate the presence of single- and four-copy homologous genes, respectively. Table S1: Transcriptome sequencing and assembly of *Sanghuangporus sanghuang* strain MS2. Table S2: Putative genes involved in the pathway of terpenoid backbone biosynthesis in *Sanghuangporus sanghuang* strain MS2. Table S3: Putative genes involved in the pathway of sesquiterpenoid and triterpenoid biosynthesis in *Sanghuangporus sanghuang* strain MS2. Table S4: Putative genes involved in the pathway of biosynthesis of polysaccharides (starch and sucrose metabolism) in *Sanghuangporus sanghuang* strain MS2. Table S5: Putative genes involved in the pathway of uridine diphosphate glucose biosynthesis in *Sanghuangporus sanghuang* strain MS2. Table S6: Putative genes involved in the pathway of ubiquinone and other terpenoid quinone biosynthesis in *Sanghuangporus sanghuang* strain MS2. Table S7: Putative genes involved in the pathway of steroid biosynthesis in *Sanghuangporus sanghuang* strain MS2. Table S8: Putative genes involved in the pathway of phenylpropanoid biosynthesis in *Sanghuangporus sanghuang* strain MS2. Table S9: Identification of cytochrome P450 genes in MS2_V1 of *Sanghuangporus sanghuang* strain MS2. Table S10: Identification of cytochrome P450 genes in MS2_V2 of *Sanghuangporus sanghuang* strain MS2. Table S11: Identification of cytochrome P450 genes in transcriptome annotation of *Sanghuangporus sanghuang* strain MS2.
