# Supplementary figures and images for: Genome Re-Annotation and Transcriptome Analyses of Sanghuangporus sanghuang
Source: J Fungi (Basel). 2023 Apr 23;9(5):505. doi: 10.3390/jof9050505 (PMC10219462; doi:10.3390/jof9050505)

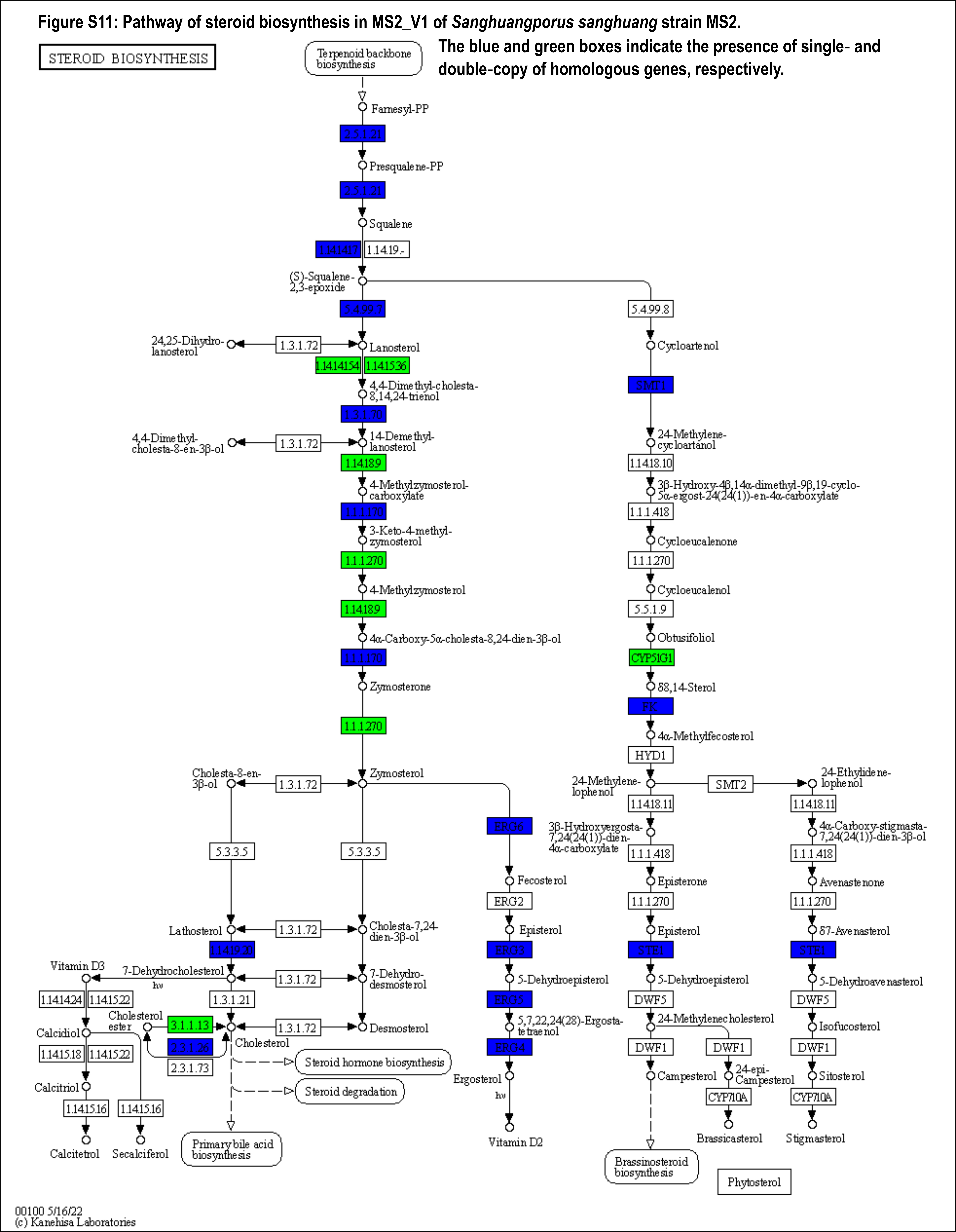

Supplement: Supplementary file 1 [file jof-09-00505-s001.zip › Supplementary Figure S11.jpg]

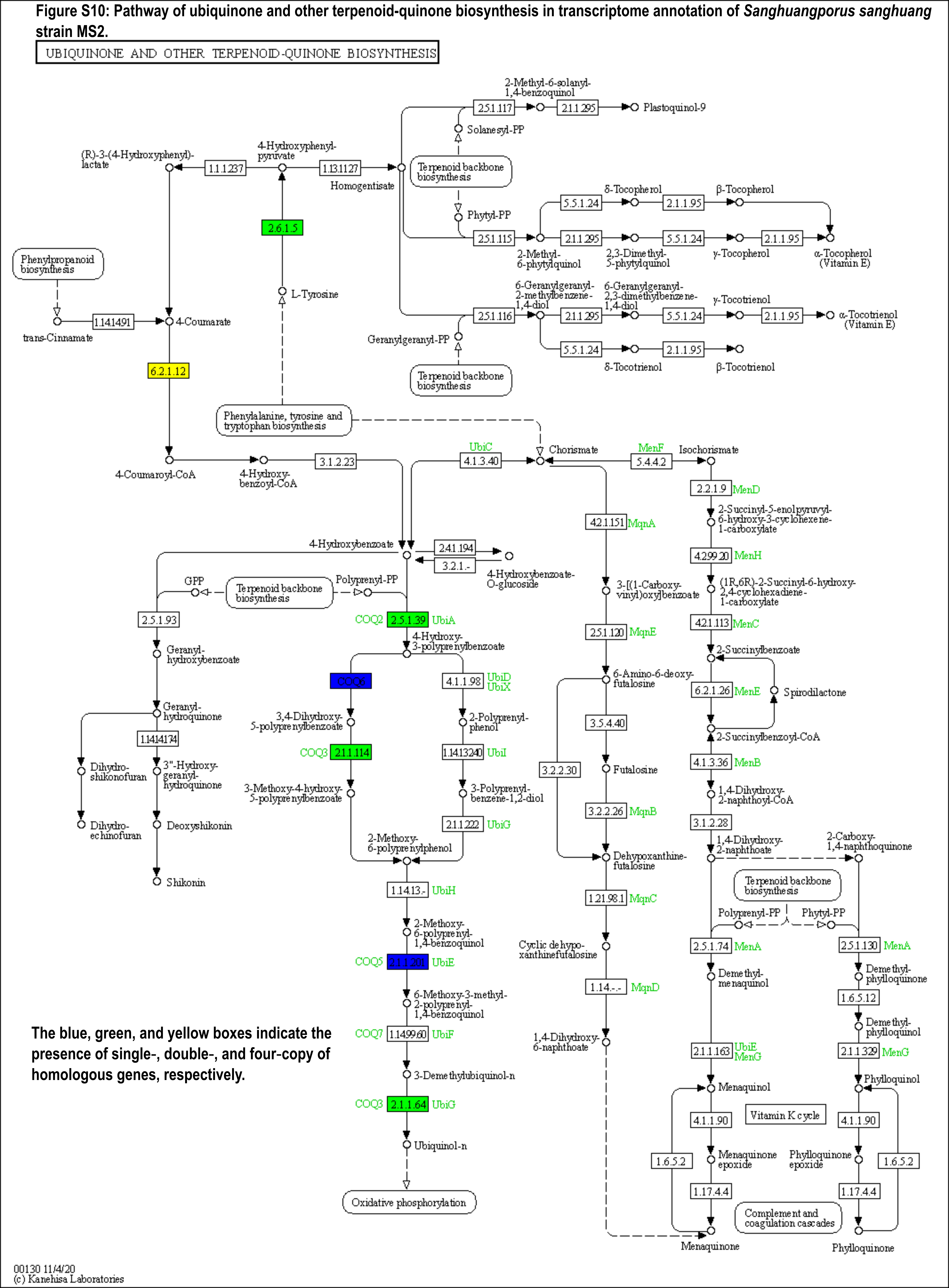

Supplement: Supplementary file 1 [file jof-09-00505-s001.zip › Supplementary Figure S10.jpg]

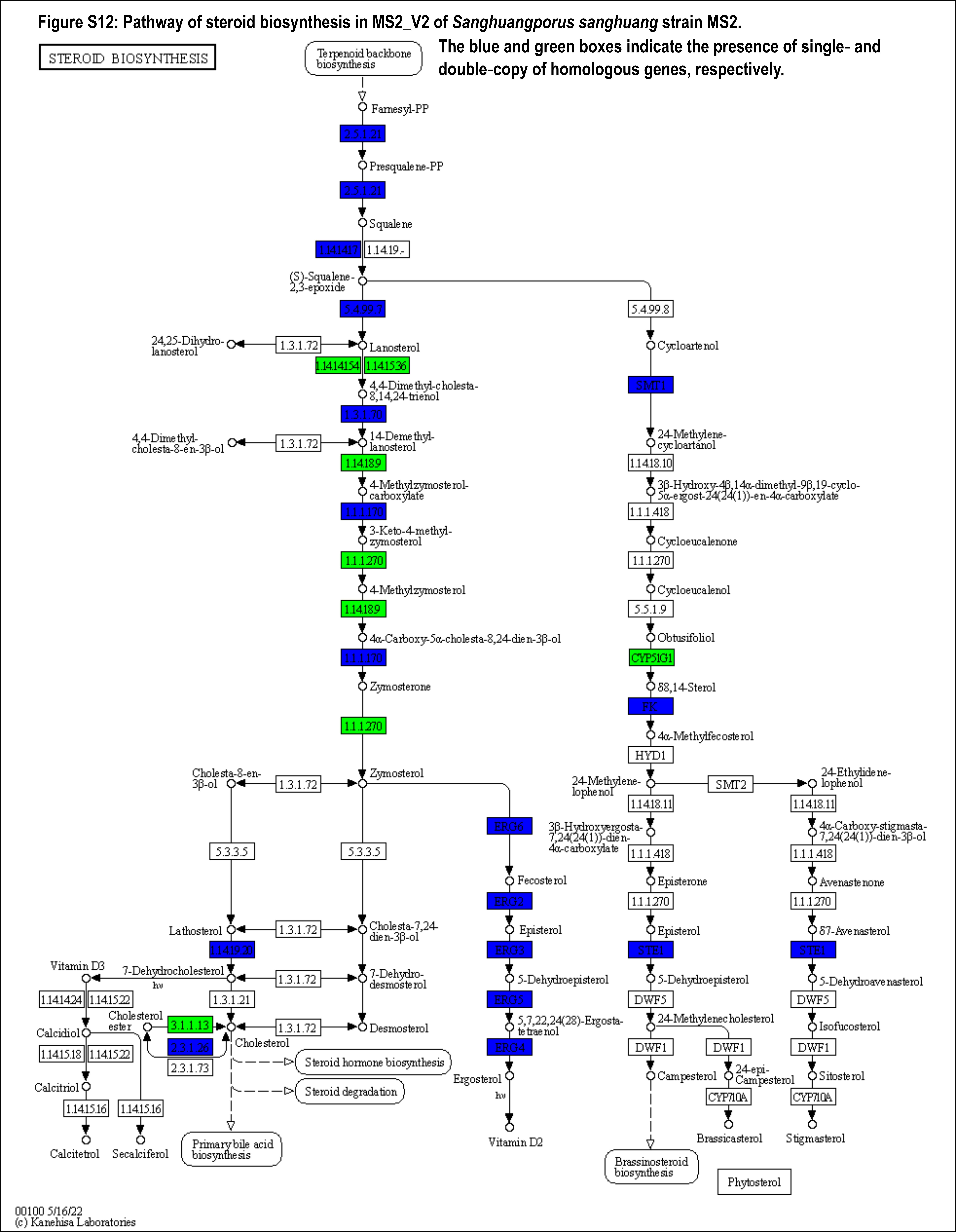

Supplement: Supplementary file 1 [file jof-09-00505-s001.zip › Supplementary Figure S12.jpg]

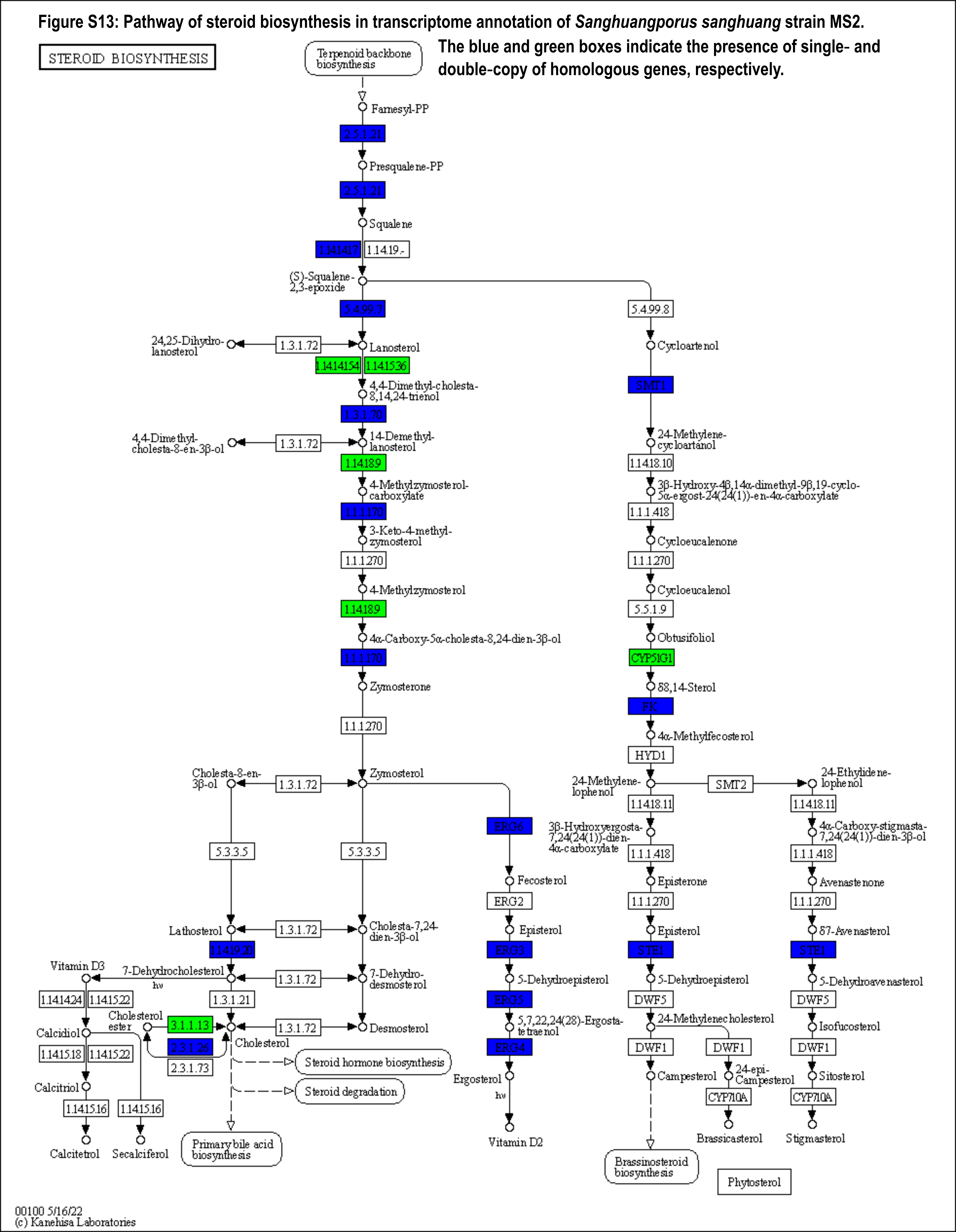

Supplement: Supplementary file 1 [file jof-09-00505-s001.zip › Supplementary Figure S13.jpg]

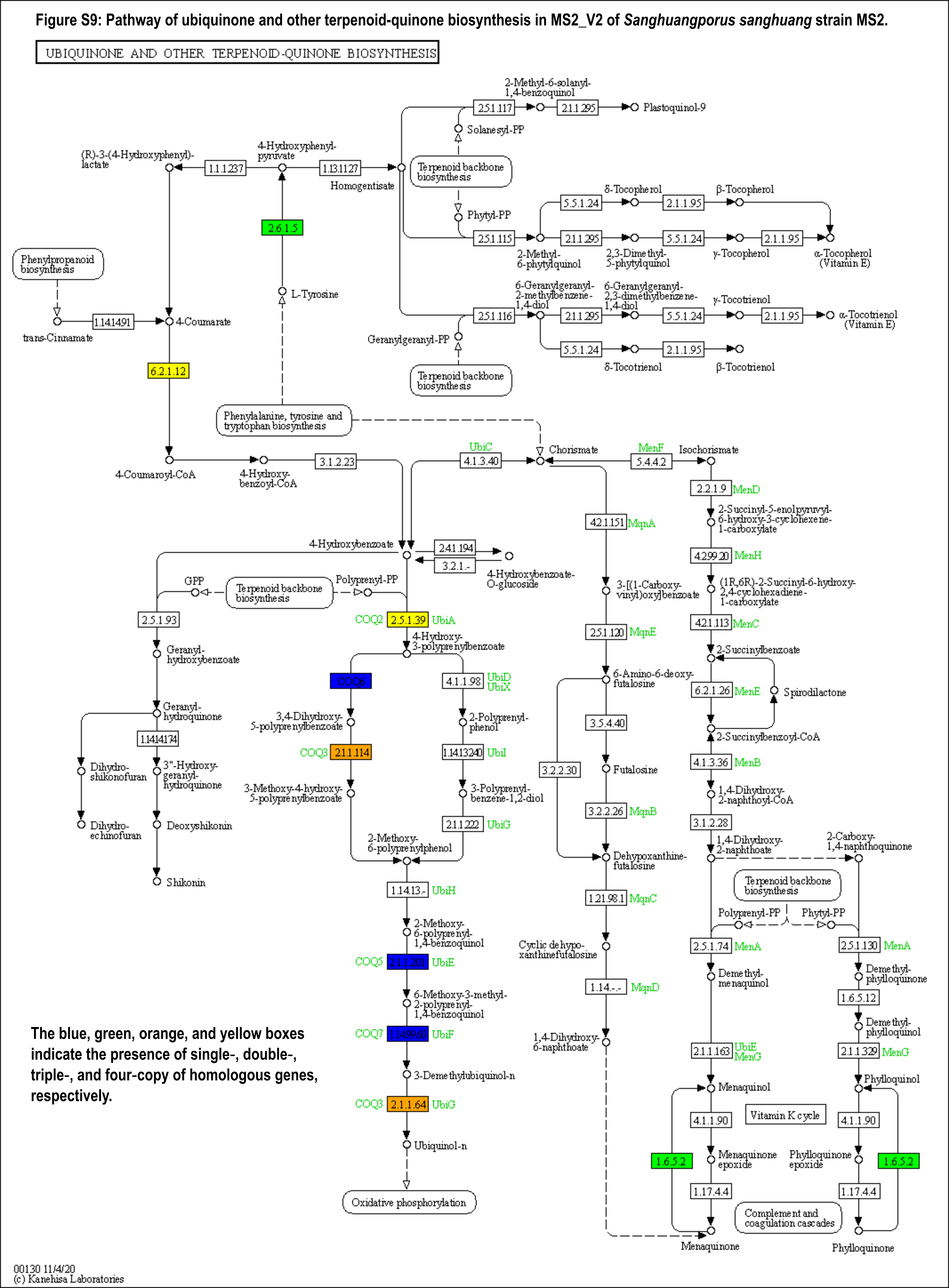

Supplement: Supplementary file 1 [file jof-09-00505-s001.zip › Supplementary Figure S9.jpg]

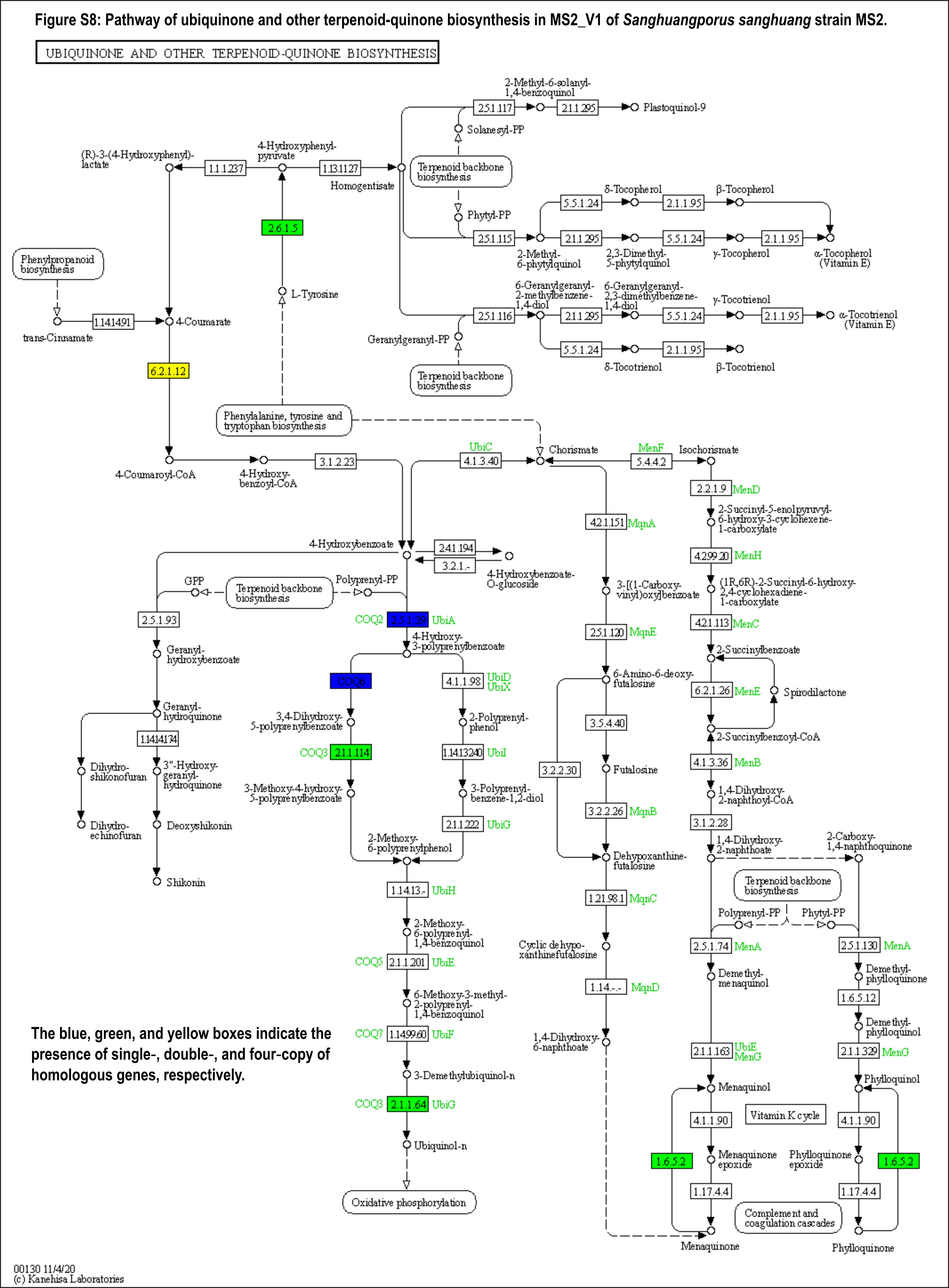

Supplement: Supplementary file 1 [file jof-09-00505-s001.zip › Supplementary Figure S8.jpg]

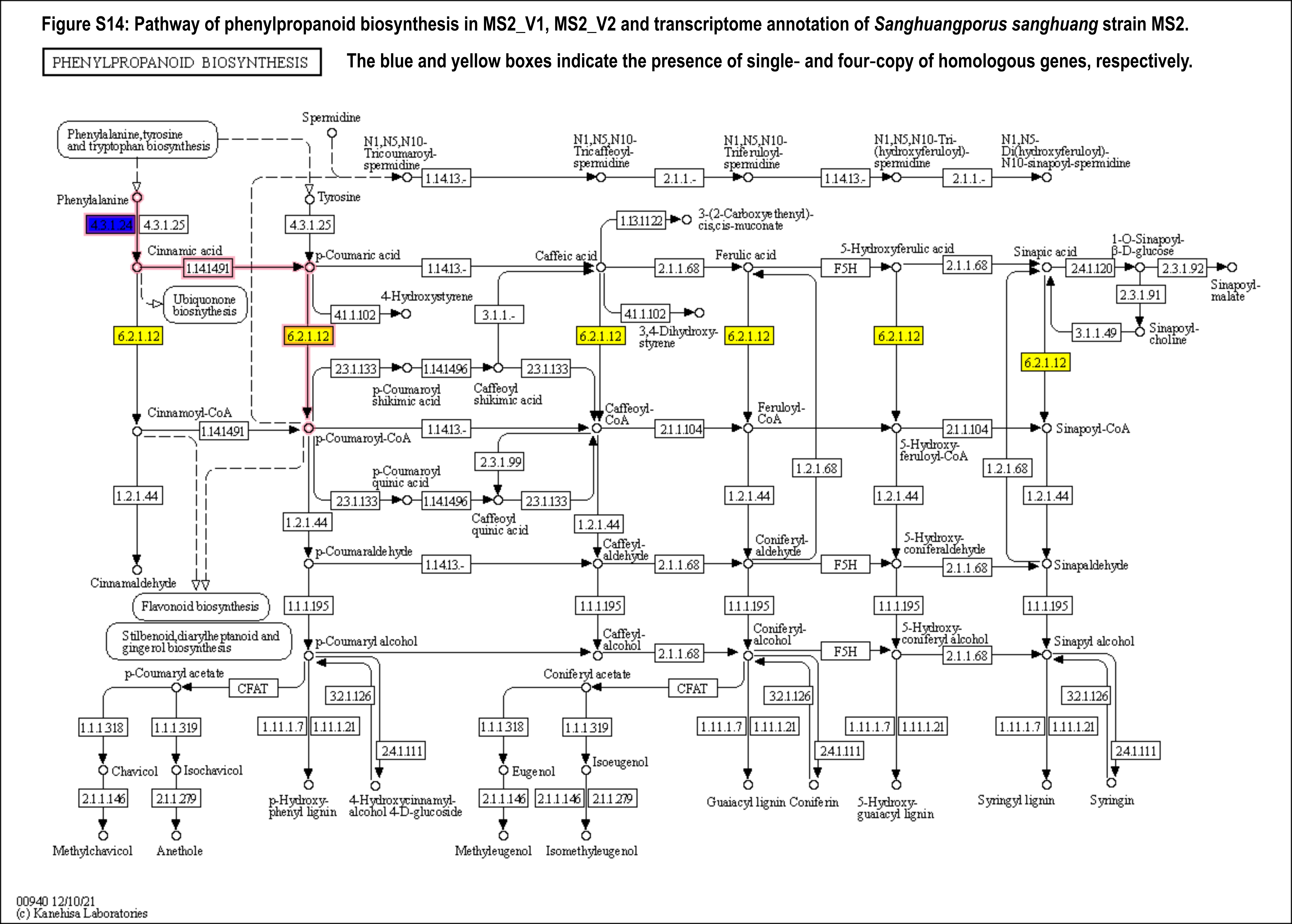

Supplement: Supplementary file 1 [file jof-09-00505-s001.zip › Supplementary Figure S14.jpg]

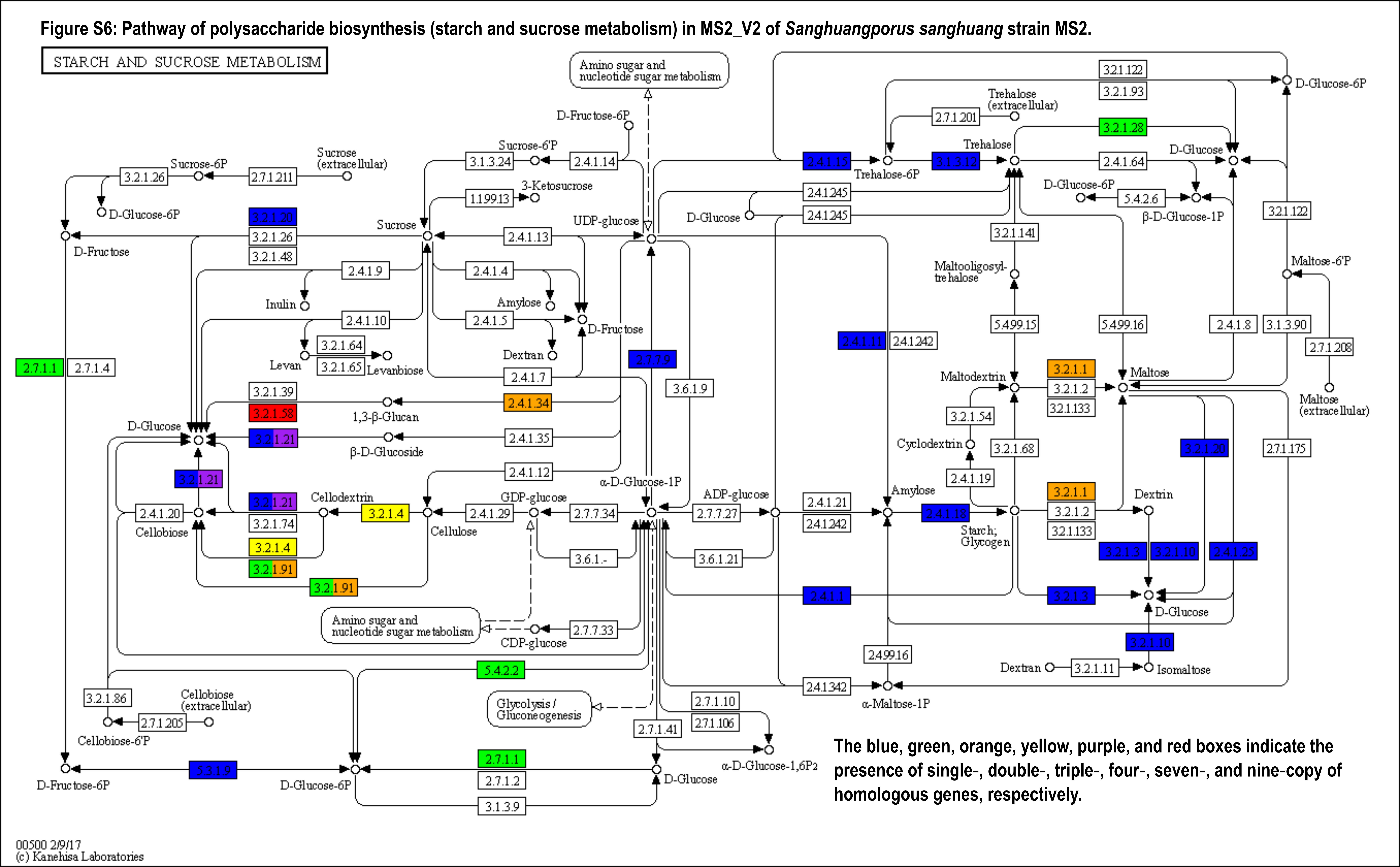

Supplement: Supplementary file 1 [file jof-09-00505-s001.zip › Supplementary Figure S6.jpg]

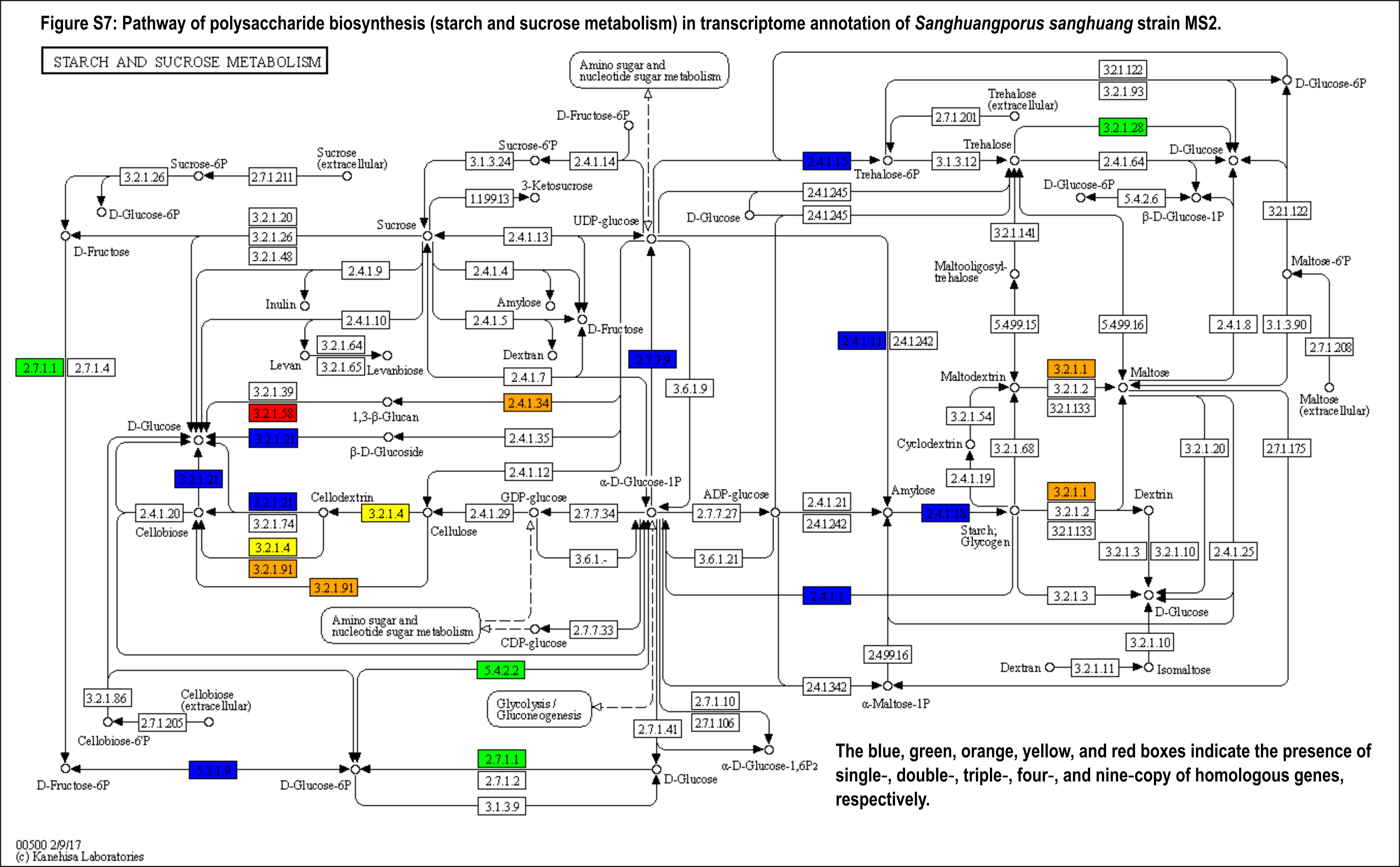

Supplement: Supplementary file 1 [file jof-09-00505-s001.zip › Supplementary Figure S7.jpg]

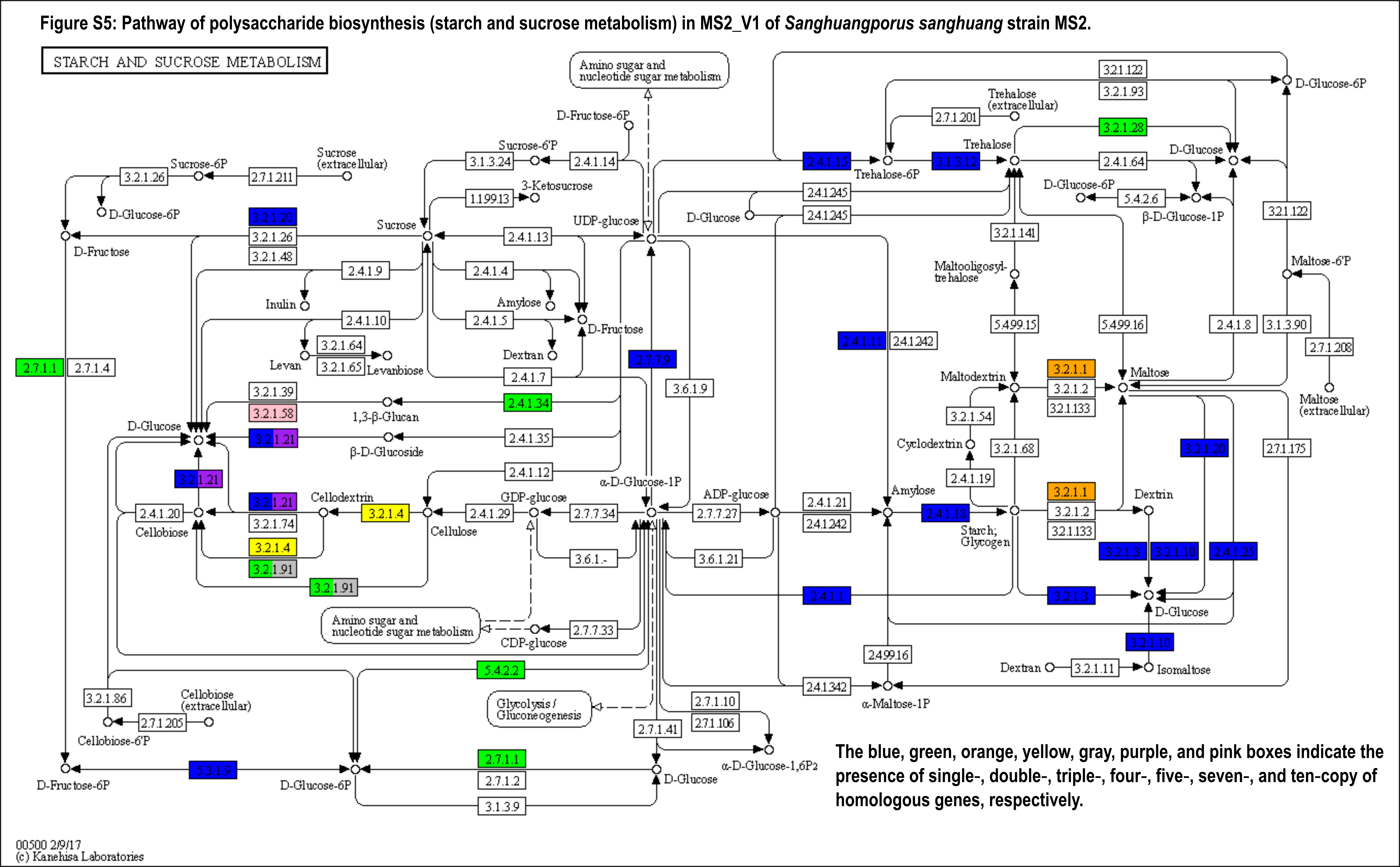

Supplement: Supplementary file 1 [file jof-09-00505-s001.zip › Supplementary Figure S5.jpg]

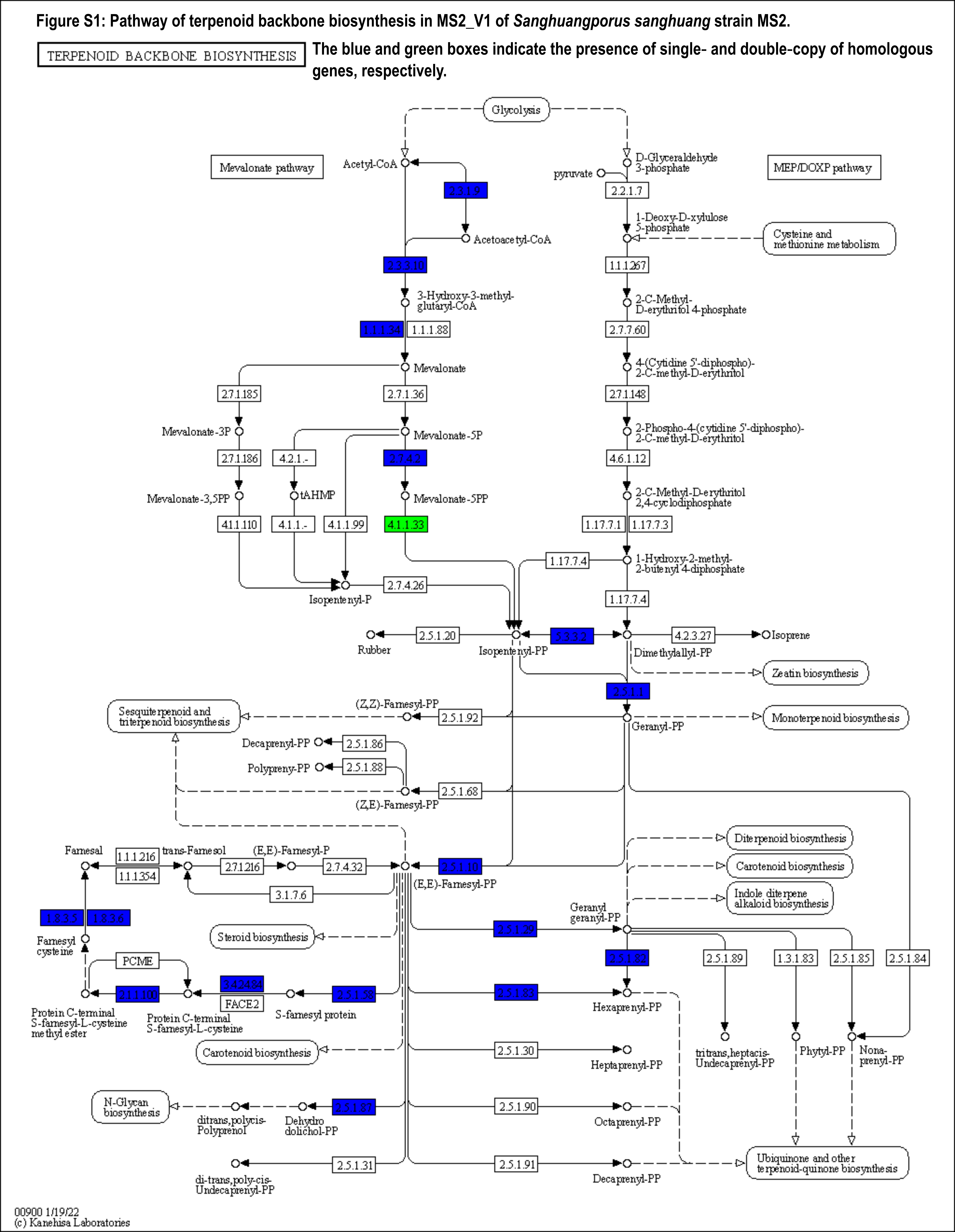

Supplement: Supplementary file 1 [file jof-09-00505-s001.zip › Supplementary Figure S1.jpg]

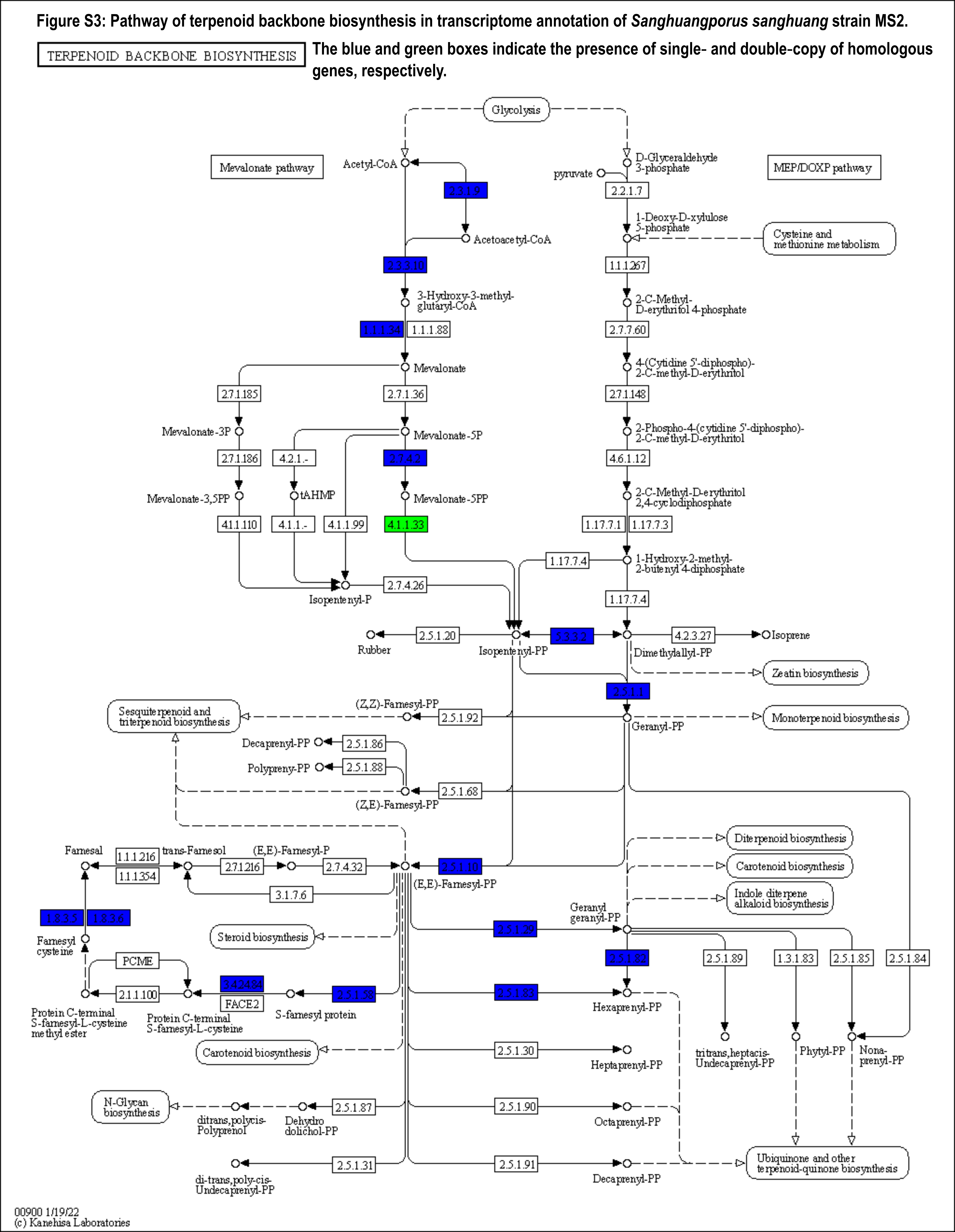

Supplement: Supplementary file 1 [file jof-09-00505-s001.zip › Supplementary Figure S3.jpg]

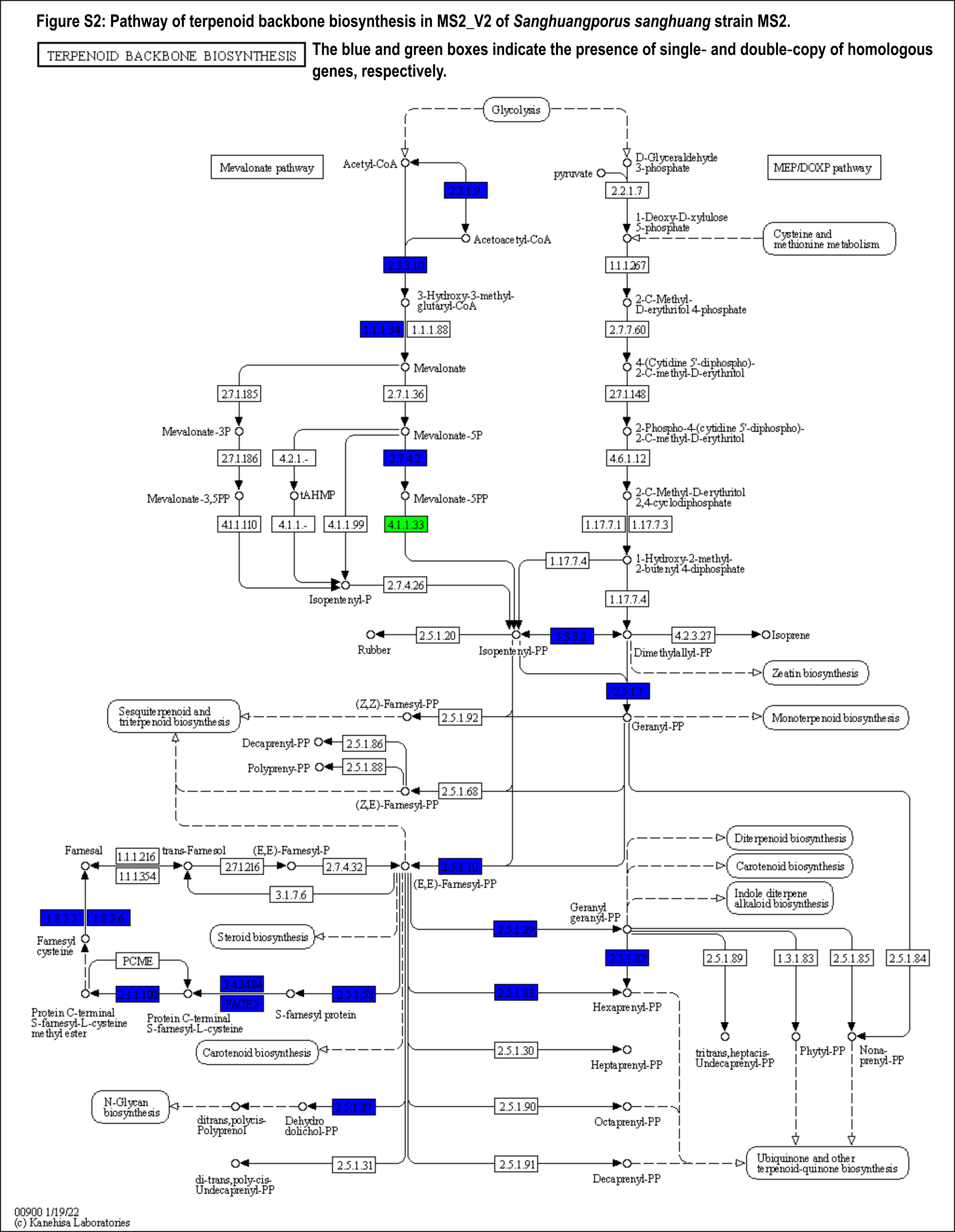

Supplement: Supplementary file 1 [file jof-09-00505-s001.zip › Supplementary Figure S2.jpg]
